# Exploring the effect of nicotine pouches on users’ health in Saudi Arabia: A cross-sectional study

**DOI:** 10.18332/tid/203510

**Published:** 2025-05-09

**Authors:** Emadeldin M. Elsokkary, Fahad A. Alsabhan, Abdulellah A. Alyahya, Saud A. Alsahli, Abdullah M. Almousa, Jehad A. Aldali, Glowi Alasiri

**Affiliations:** 1 Department of Psychology, College of Social Sciences, Imam Mohammad Ibn Saud Islamic University, Riyadh, Saudi Arabia; 2 College of Medicine, Imam Mohammad Ibn Saud Islamic University, Riyadh, Saudi Arabia; 3 Department of Pathology, College of Medicine, Imam Mohammad Ibn Saud Islamic University, Riyadh, Saudi Arabia; 4 Department of Biochemistry, College of Medicine, Imam Mohammad Ibn Saud Islamic University, Riyadh, Saudi Arabia

**Keywords:** nicotine, pouches, awareness, symptoms, Saudi Arabia

## Abstract

**INTRODUCTION:**

Tobacco consumption is a significant public health concern and a worldwide avoidable contributor to mortality. Various alternatives have been developed to either support cessation or mitigate the impact of new tobacco products entering the market in order to address the issue. One such recent alternative is the utilization of nicotine pouches. The objective of this study is to investigate the symptoms and awareness associated with the use of nicotine pouches in Saudi Arabia.

**METHODS:**

A cross-sectional online survey was distributed in both Arabic and English using Google Forms, and it was conducted between 11 January and 4 March 2024 among the general population residing in the Riyadh province of Saudi Arabia. Statistical analysis was conducted using SPSS Version 27, with a p<0.05 indicating significance.

**RESULTS:**

A total of 385 participants were included in the study. Married individuals were significantly more likely to have used nicotine products than unmarried (76.2% vs 32.4%, p<0.001). Males were much more likely than females to have used nicotine products (63.1% vs 7.3%, p<0.001). More non-Saudis appeared to have used nicotine products compared to Saudis (57.1% vs 36.0%, p<0.001), while an age-related pattern in the usage of nicotine products, particularly among those aged 30–35 years (90.9%) was noted. In terms of nicotine pouch usage, 79 participants (73.1% vs 26.9%, p<0.001) reported having used nicotine pouches. Symptoms such as mouth or gum irritation and nausea were more common, but no specific pattern emerged.

**CONCLUSIONS:**

Within our sample, daily was the most common frequency of current nicotine pouch usage, while nausea and mouth or gum irritation were reported more frequently.

## INTRODUCTION

One of the primary contributors to a diverse range of human health issues is smoking, which encompasses a spectrum of illnesses ranging from life-threatening cardiovascular diseases to debilitating lung cancers and chronic obstructive pulmonary disease^1^. The detrimental consequences of smoking extend beyond these circumstances, affecting nearly every organ in the human body^2^. Cigarette smoking remains the primary method of nicotine consumption in a wide range of demographics and regions^3^. Nicotine, a chemical compound that is naturally present in tobacco leaves, is the primary cause of the addictive nature of cigarette smoking. Nicotine is eventually released into cigarette smoke during the combustion process. The addictive appeal of smoking is significantly enhanced by the natural prevalence of nicotine in the tobacco plant, which captivates users with its potent psychoactive effects^4^. The effects of nicotine are rapidly disseminated throughout the duration of the frame as it rapidly and effectively permeates the bloodstream upon inhalation during a smoking session^5^. Nicotine binds to neuronal nicotinic receptors, which may help regulate mood and promote relaxation^6^, while the pharmacokinetic profile of nicotine shows a sudden rise in plasma nicotine levels followed by a decrease in those levels^7^.

Recently, a brand-new category of nicotine products, known as nicotine pouches (NPs), has come into existence. NPs have a physical resemblance to Swedish snus, but they do not contain any tobacco leaf, but they do contain nicotine salts. Oral nicotine pouches, also known as ONPs, are a growing variety of smokeless nicotine products that are currently in the process of becoming more popular^8^. Nicotine pouches typically comprise small, white pouches that measure approximately 25 mm in length. To facilitate the gradual release of nicotine without the need for combustion, these pouches are intended to be placed stealthily between the gums and the lips in order to maintain their discretion^9^. The contents of these pouches contain a wide variety of components that have been meticulously chosen. In addition to nicotine, which is the primary active component, nicotine pouches may also contain a variety of flavors, ranging from mint and citrus to more individual fruit blends, to cater to a wide range of user interests and preferences^10^. Furthermore, alkaline salts are frequently incorporated into the components to adjust the pH level of the contents of the pouch. This adjustment helps to maximize the amount of nicotine that is absorbed and improves the overall effectiveness of the product^10^. This may increase attractiveness and lead to an increase in nicotine consumption, particularly among populations that were previously unfamiliar with nicotine, such as young people and teenagers^11^.

In 2023, nicotine pouches were introduced in Saudi Arabia and as a result there has been a sense of both interest and challenge regarding the potential effects that they may have on users^12^. According to our hypothesis, consumers of nicotine pouches might experience gum irritation and gastroesophageal reflux disorders. To verify our hypothesis, we investigate the effects that nicotine pouches have on the health of users in Saudi Arabia.

## METHODS

### Study design and settings

This cross-sectional study was conducted at the Department of Pathology, College of Medicine, Imam Muhammad bin Saud Islamic University, Riyadh, Saudi Arabia.

### Study participants and sample size calculation

The general population of the Riyadh province of Saudi Arabia comprised the study participants. The committee of the Institutional Review Board (IRB) at Imam Mohammad Ibn Saud Islamic University reviewed and approved this research (project number 625–2024, dated 20 March 2024). The study sample size was calculated using power calculator Raosoft. Riyadh province has a population of approximately 8 million. A sample size of 385 participants was deemed adequate to achieve a 95% confidence level with a 5% margin of error. Nevertheless, 385 individuals participated in the current study.

### Study questionnaire development

This study conducted an online survey on Google Forms in Arabic and English, in the Kingdom of Saudi Arabia between 21 March and 28 June 2024. The survey purposely targeted individuals who have used nicotine pouches to report any side effects experienced after their use. The survey was distributed via social media platforms (WhatsApp and Telegram) and places where nicotine pouches were sold (supermarkets and tobacco stores). Participants were required to provide electronic consent before participating. The first section of the survey collected demographic information (gender, age, nationality, marital status). The second section of the survey collected participants’ types of previous nicotine products, nicotine dosage, and brand of the pouches. The third section of the survey collected participants who reported side effects experienced after using the pouches.

### Statistical analysis

The statistical analysis was performed using the Statistical Package for Social Sciences (SPSS) Version 27 (IBM, Armonk, New York, NY, USA). Descriptive statistics were used to summarize qualitative data, such as nicotine pouch usage and related symptoms. The Pearson chi-squared test was applied to assess the relationships between categorical variables, such as demographic factors (e.g. gender, age, marital status, and nationality) and nicotine usage patterns. This test evaluated whether significant associations existed between these variables. Chi-squared test for differences was used to examine differences in the usage patterns (e.g. dosage, frequency, and brand preference) across various groups. This test helped identify whether significant differences existed between the groups based on their nicotine pouch consumption behaviors. A p<0.05 was considered statistically significant.

## RESULTS

Table 1 showed that married individuals were significantly more likely to have used nicotine products (76.2% vs 32.4%, p<0.001), suggesting a strong relationship between being married and nicotine use. Males were much more likely to have used nicotine products (63.1% vs 7.3%, p<0.001), highlighting a notable gender difference in nicotine use. There was a borderline significant association between nationality and nicotine use (p<0.051). Non-Saudis appeared to have used nicotine products more often (57.1% vs 36.0%, p<0.001). There was a clear age-related pattern in the usage of nicotine products, with younger age groups (15–23 years) being less likely to use these products (31.4%) compared to older age groups, particularly those aged 30–35 years (90.9%).

**Table 1:** Usage of nicotine products among various demographic groups in Saudi Arabia (N=385)

*Characteristics*	*Categories*	*Have you ever used a nicotine* *product, even once?*	
		*Yes* *n (%)*	*No* *n (%)*
**Total**		143 (37.1)	242 (62.9)
**Marital status**	Married	32 (76.2)	10 (23.8)
	Unmarried	111 (32.4)	232 (67.6)
**Gender**	Male	130 (63.1)	76 (36.9)
	Female	13 (7.3)	166 (92.7)
**Nationality**	Saudi	131 (36.0)	233 (64.0)
	Non-Saudi	12 (57.1)	9 (42.9)
**Age** (years)	15–23	95 (31.4)	208 (68.6)
	23–30	32 (54.2)	27 (45.8)
	30–35	10 (90.9)	1 (9.1)
	35–40	3 (60.0)	2 (40.0)
	>40	3 (42.9)	4 (57.1)

Table 2 shows that 108 participants (75.5% vs 24.5%, p<0.001) reported being aware of nicotine pouches before the study, while 79 participants (73.1% vs 26.9%, p<0.001) reported having used nicotine pouches. The most common number of nicotine pouches used per day was <5 units (46.8%), followed by 5–10 units (30.4%), and fewer participants reported using >10 units (p<0.001). The most commonly reported dosage was 6–10 mg (55.7%), with smaller percentages using 1–3 mg or 3–6 mg (both 10.1%, p<0.001). The most commonly used brand was DRZT (88.6%), while fewer participants reported using VELO or other brands (p<0.001).

**Table 2:** Difference between awareness, usage, dosage and symptoms associated with nicotine pouch use

*Item*	*Categories*	*n*	*χ* ^2^	*p*
**Were you already aware of nicotine pouches before this study?**	Yes	108	37.266	<0.001
	No	35		
	Total	143		
**Have you ever used nicotine pouches?**	Yes	79	23.148	<0.001
	No	29		
	Total	108		
**During the days that you use nicotine pouches, how many units do you use?**	<5	37	51.063	<0.001
	5–10	24		
	11–15	7		
	16–20	6		
	>20	5		
	Total	79		
**What dosage (mg) are the nicotine pouches you use?**	1–3	8	43.785	<0.001
	3–6	8		
	6–10	44		
	>10	19		
	Total	79		
**What brand of nicotine pouches you use?**	DZRT	70	108.633	<0.001
	VELO	4		
	Other	5		
	Total	79		
**Do you suffer from any of these symptoms?**	No symptoms	27	4.071	0.396
	Mouth or gum irritation	33		
	Nausea	33		
	Gastroesophageal reflux	28		
	High heart rate	20		
	Total	141		

Significant differences, p<0.05.

While symptoms such as mouth or gum irritation and nausea were more frequently reported, no notable pattern of symptom distribution was evident.

## DISCUSSION

The study aimed to describe the awareness and use of nicotine pouches, both in general and according to tobacco product usage, as well as to characterize the symptoms associated with nicotine pouch use and other behaviors among individuals who use nicotine pouches. This study indicates that men, particularly married individuals, frequently use a dosage of 6–10 mg per day. Additionally, the most common symptoms reported by nicotine pouch users varied, with mouth or gum irritation and nausea being the most prevalent.

Recently, a new type of nicotine product, known as oral nicotine pouches, has emerged. These pouches are smokeless and tobacco-free but contain nicotine salts^8^. They consist of small pouches approximately 25 mm in length that are placed between the gums and lips, allowing nicotine to be absorbed into the bloodstream. This offers a method of nicotine delivery that does not involve inhaling smoke^13^. The findings of this study reveal that the sociodemographic characteristics of nicotine users are mostly males, married, aged ≥23 years. This is consistent with the 2019 Global Adult Tobacco Survey (GATS) conducted by MOH in Saudi Arabia^13^ and several other studies involving nicotine products and their prevalence in Saudi Arabia^14-16^. A significant difference between our study and another^8^, which uses data from the 2013 SHIS, is in the prevalence of tobacco products based on marital status, while our findings revealed a higher prevalence of nicotine products in married participants similar to more recent studies^16,17^, the other study reported unmarried participants to be significantly more likely to use nicotine products. Of note, regarding our study and those previously mentioned, only one study^17^ is nationally representative.

As for the usage of nicotine pouches, excluding participants with no previous use of nicotine products or any awareness of nicotine pouches, 73.1% of participants aware of nicotine pouches have ever used them. Comparatively, the finding of Sparrock et al.^18^ investigating awareness and use of nicotine pouches among a 2021 nationally representative sample of US adult current commercial tobacco users, found that 39.2% of those aware of nicotine pouches have ever used NPs. Another study^19^, conducted in 2019 among UK current and former smokers and vapers, shows only 27.8% of participants that are aware of NPs, have ever used them.

The daily use of nicotine pouches among consumers suggests a potential dependence, consistent with findings from studies on electronic cigarettes conducted by Soule et al.^20^ which also showed a dependence similar to that of traditional cigarettes. Also, some consumers might be using nicotine pouches to quit smoking by substituting cigarettes with them. Similar findings were observed in research done by Benmarhnia et al.^21^ on electronic cigarettes used for smoking cessation, where daily users began to use ENDS (Electronic Nicotine Delivery Systems) to quit smoking. Furthermore, consumers might replace cigarettes with nicotine pouches due to the perception that they are less harmful. Prior research by Plurphanswat et al.^22^ indicated that consumers switched from tobacco products to ZYN pouches because they perceived them to be less harmful.

Consumers showed a tendency to prefer higher dosages with fewer units, suggesting they may seek a particular level of satisfaction. On the other hand, some opted for frequent usage of low to normal dosages, implying they may not have been satisfying an addiction but were still consuming comparable nicotine levels to traditional tobacco products. Research by Stanfill et al.^9^ has found that nicotine and pH levels in some nicotine pouches are similar to those in conventional tobacco products. Moreover, frequent consumption of large amounts of nicotine pouches could be indicative of developing a negative behavior, like binge eating, where individuals consume large quantities while feeling powerless to stop, accompanied by discomfort and shame. A study conducted by Anzengruber et al.^23^ on eating disorders involving 1524 women found an increased risk of smoking among those who engaged in binge eating behaviors.

The data also show some interesting patterns regarding symptoms. As nicotine pouches are used in NRT, it is expected for them to be less harmful than cigarettes^24,25^. While almost a third (34.2%) reported no issues, others experienced mouth or gum irritation (42%) and nausea (42%). This is to be expected, as both nausea and mouth irritations are common effects of nicotine use in general^5^. Interestingly, one study done by Mallock-Ohnesorg et al.^26^ in which the oral mucosa was inspected 10 minutes after the removal of nicotine pouches, moderate mouth irritation was reported by participants, local adverse effects were seen even with nicotine-free pouches, while mouth irritation during smoking was low. This indicates that it may be possible for mouth irritation to be due to other ingredients in the pouches^26^. Similarly, 35.4% of users reported gastroesophageal reflux (GERD) and 25.3% experienced high heart rate. While these could be coincidental, both symptoms are known to be correlated or induced by tobacco products^27,28^.

### Limitations

This cross-sectional study conducted in Saudi Arabia to investigate the impact of nicotine pouches on users has several limitations that should be taken into account. Initially, the cross-sectional design precludes the establishment of causality between the use of nicotine pouches and the observed outcomes, thereby restricting the study to associations rather than definitive conclusions. Furthermore, the accuracy of the findings may be compromised by the introduction of biases, such as recall bias or social desirability bias, as a result of the reliance on self-reported data. Another concern is residual confounding, which occurs when not all potential confounders have been considered, potentially resulting in biases in the observed associations. Additionally, the findings’ robustness is restricted by the absence of adjusted comparisons between various user groups. Moreover, the results may have limited generalizability to other populations or countries, as the study is context-specific to Saudi Arabia, and the effects of nicotine pouches elsewhere could be influenced by cultural, social, and regulatory differences. In addition, it is important to note that our study is subject to selection bias as the survey was administered online which may exclude people without internet connection or those who are not social media users, which may limit the generality of the sample. Self-selection bias is also possible as those who took the time to respond may have had more intense opinions or stories regarding nicotine pouch use, which may have biased the results.

## CONCLUSIONS

This study on the effect of nicotine pouches on users in Saudi Arabia revealed that some users reported symptoms like mouth or gum irritation and nausea more frequently. Further research is needed to explore in depth the factors associated with use and the appearance of adverse effects in the Saudi population.

## Data Availability

The data supporting this research are available from the authors on reasonable request.
